# Newborn Screening: Equity for Aboriginal and Torres Strait Islander Families in the Context of Emerging Genomics

**DOI:** 10.5694/mja2.70272

**Published:** 2026-09-02

**Authors:** Gail Garvey, Sarah Norris, Joanne Scarfe, Louise Lyons

**Affiliations:** ^1^ First Nation Cancer and Wellbeing Program, School of Public Health The University of Queensland Herston Queensland Australia; ^2^ Leeder Centre for Health Policy, Economics and Data, School of Public Health The University of Sydney Sydney New South Wales Australia; ^3^ ALIGN Network The Kids Research Institute Australia Nedlands Western Australia Australia

**Keywords:** genetic testing, indigenous health, maternal health

## Abstract

Australia's newborn bloodspot screening (NBS) program is offered to every newborn. It screens for 34 rare conditions with the potential for hundreds more to be added using genomics. Despite NBS being available in Australia since the 1960s, there is a lack of evidence regarding the participation and experiences of Aboriginal and Torres Strait Islander peoples in NBS. As Australia considers a future where genomics might be used in NBS, there is a critical window of opportunity to understand and prioritise the perspectives, hopes and fears of Aboriginal and Torres Strait Islander peoples regarding the utility of genomics in NBS.

## 1 The Newborn Bloodspot Screening (NBS) Program and Matters of Equity

### 1.1 Positionality of the Authors

Our team acknowledges the importance of reflexively considering our diverse backgrounds, perspectives and values. Gail Garvey is a proud Kamilaroi woman and senior researcher, and Louise Lyons is a proud Jaadwa woman and Traditional Owner from Western Victoria; both have expertise in Aboriginal health, research and genomics. Sarah Norris is a non‐Indigenous researcher with expertise in health technology assessment and health policy, and Joanne Scarfe is a non‐Indigenous health economist and health services researcher.

The CONSIDER reporting criteria checklist for health research involving Indigenous peoples was completed for this article and can be found in the Supporting Information [1], together with an ethical research statement.

## 2 Background

The newborn bloodspot screening (NBS) program is one of Australia's most successful population health initiatives, with 99% national participation. NBS programs are administered independently by states and territories and supported through the National Health Reform Agreement [2]. Over 300,000 newborns are screened each year, enabling early identification of rare genetic conditions and metabolic disorders. Approximately 1 in 1000 newborns is identified with a genetic condition that may have otherwise been undetected until symptoms emerged. NBS facilitates early diagnosis, timely treatment and support to be provided for individuals with screened conditions, thereby improving outcomes for the child and their family [2].

Despite high participation rates overall, access to mainstream screening programs such as NBS is not always equitable or culturally safe for Aboriginal and Torres Strait Islander peoples. Currently, there are no data regarding the 1% of newborns that are not screened. Data on uptake, refusal rates, follow‐up completion and lived experiences of Aboriginal and Torres Strait Islander peoples within the NBS program are notably absent, as are data on the rate of Aboriginal and Torres Strait Islander infants diagnosed with NBS‐detected rare conditions. NBS program inequalities for First Nations peoples include, but are not limited to, jurisdictional variation, an absence of disaggregated data on screening and program outcomes, culturally safe information and consent processes and a lack of First Nations nurses, midwives and genetic counsellors. The Western Australia NBS program appears to be the only Australian program that publicly reports NBS participation and outcome data; however, the data are not disaggregated by Aboriginal or Torres Strait Islander status [3]. These data gaps limit our ability to assess and address potential disparities in participation and benefit from the NBS program.

Regarding the promised expansion of the NBS to include more conditions and ensure consistency of programs across Australia, public consultation undertaken in 2022 identified many issues that should be considered [4]. The National Aboriginal Community Controlled Health Organisation (NACCHO) noted that the NBS program has the potential to positively contribute towards the National Agreement target that ‘Aboriginal and Torres Strait Islander children thrive in their early years*’* and that ‘by 2031, increase the proportion of Aboriginal and Torres Strait Islander children assessed as developmentally on track in all five domains of the Australian Early Development Census (AEDC) to 55%*’* [5]. NACCHO further commented that this target could only be met if screening is culturally safe and accessible for all Aboriginal and Torres Strait Islander children and their families [5].

For newborns who are screened, timely and culturally safe follow‐up and treatment are essential. However, systemic inequities within the Australian healthcare system continue to present significant challenges for Aboriginal and Torres Strait Islander peoples, including those living in rural and remote areas where access to specialist services, care and treatment may be limited. Collaboration and shared responsibility across primary and tertiary health sectors (e.g., Aboriginal Community Controlled Health Organisations, birthing centres and hospitals) is required to ensure continuity of care and communication within appropriate timelines for follow‐up and responding to any critical conditions identified from participation in NBS [5]. For many Aboriginal and Torres Strait Islander people and families, generic mainstream information often fails to meet their cultural and linguistic needs [6], which may lead to increased fear and mistrust about what is being done to their baby and why and how the results will be used. Providing Aboriginal and Torres Strait Islander parents and families with culturally appropriate NBS information is a fundamental aspect of patient‐centred care [7], and yet, to our knowledge, there is a lack of culturally appropriate and safe NBS resources available, despite being recommended in the 2018 *NBS National Policy Framework* [8]. The lack of these resources, along with a limited Indigenous nursing and midwifery workforce, and the jurisdictional variation in how families receive information about NBS raises concerns about the processes used to obtain informed consent from Aboriginal and Torres Strait Islander peoples. The importance of addressing these gaps should be understood within the broader context of health inequities experienced by Aboriginal and Torres Strait Islander peoples such as systematic racism and other factors that drive inequitable access to health services. We propose that the development and use of high‐quality, culturally responsive health education resources that are consistent across Australia is essential to supporting improved health knowledge, informed decision‐making and equitable health gains.

## 3 NBS and the Emergence of Genomics

Emerging technologies, such as genomic sequencing, can offer the opportunity to substantially expand NBS by detecting a wider range of rare conditions [9, 10]. Implementation of genomic sequencing raises ethical and practical concerns such as the interpretation of genetic variants, including uncertainty regarding genotype–phenotype relationships and disease penetrance. These challenges may compromise timely, accurate diagnosis of a health condition for populations under‐represented in genome reference databases, with implications for diagnostic follow‐up, clinical monitoring, access to genetic counselling and long‐term care [10]. Further, the identification of genetic disorders in a newborn may have implications for other members of their family, requiring broader considerations than that of the child alone. Including genomics in NBS also raises important cultural concerns for Aboriginal and Torres Strait Islander peoples [11]. Many of these concerns stem from the ongoing impact of colonisation, historical and contemporary mistrust, the unethical use of biological samples [12], poor research practices and the association between genetics and eugenics [13]. There are also concerns about how genomics could be used against Aboriginal and Torres Strait Islander peoples to reinforce racial stereotypes and increase experiences of discrimination and compound existing systemic inequalities in healthcare [11, 14, 15]. The use of genomics, including in NBS, could challenge the established social and cultural criteria based on descent, self‐identification, community acceptance and cultural adoption practices [16]. Further, there may be concerns about what genomics in NBS might reveal or mean for existing family relationships (maternal and paternal) [17]. Although there is emerging evidence that Aboriginal and Torres Strait Islander peoples have some unique genetic differences [18, 19], more evidence is needed so that more accurate ways to diagnose, treat and prevent many health conditions, including rare diseases, can be achieved for Aboriginal and Torres Strait Islander peoples.

## 4 Genomics and Aboriginal and Torres Strait Islander Peoples

Genomics raises important cultural concerns for Aboriginal and Torres Strait Islander peoples that require specific actions. Central to this is developing meaningful relationships with Aboriginal and Torres Strait Islander communities through genuine engagement that respects collective decision‐making, Indigenous leadership and sovereignty. Given this, a growing number of initiatives in genomics and Aboriginal and Torres Strait Islander peoples have emerged. One such initiative is the Australian Alliance for Indigenous Genomics (ALIGN). ALIGN is a partnership between academia, industry, government and Aboriginal and Torres Strait Islander communities that seeks to build Indigenous leadership and involvement in genomics (e.g., research, precision healthcare, data sciences), including ethics and Indigenous knowledge systems to reduce health inequality among Australia's First Peoples (https://indigenousgenomics.com.au). Another initiative is the recently released *Guiding Principles: Ensuring Culturally Safe Health Genomics with Aboriginal and Torres Strait Islander Peoples* [20]. Six specific principles are included to guide genomics clinical practice and research: Aboriginal and Torres Strait Islander Peoples' Rights; Culturally Safe Genomics; Health Equity; Data Sovereignty; Informed Consent and Genomics Health Workforce, and provide a valuable framework and guidance for NBS and the use of genomics. These guiding principles [20] draw on key national agreements and frameworks, including the *National Agreement on Closing the Gap* [21] and align with international declarations on the rights of Indigenous peoples [22].

## 5 Indigenous Data Sovereignty and Governance and NBS

Indigenous data sovereignty and governance are fundamental to ensuring ethical, equitable and culturally safe use of data in NBS. Drawing on established Australian frameworks, such as the *Maiam nayri Wingara Indigenous Data Sovereignty* [23], *Making data Findable, Accessible, Interoperable and Reusable (FAIR) and CARE Principles* [24], can support the development of culturally informed and respectful NBS protocols and processes. For example, Guthrie card storage and data must be managed and used in ways that prioritise Aboriginal and Torres Strait Islander peoples' inherent rights to control the collection, ownership, storage, analysis, interpretation and application of data relating to their communities, families and individuals.

## 6 Conclusion

Australia's NBS program requires a national, culturally safe and equity‐focussed approach that privileges the perspectives and experiences of Aboriginal and Torres Strait Islander peoples and supports a culturally informed workforce to ensure equitable access and outcomes. Genomic newborn screening cannot be assumed to be equitable when baseline participation and experiences of Aboriginal and Torres Strait Islander peoples remain poorly understood.

## Author Contributions

Gail Garvey led the conceptualisation and writing of the original manuscript. All authors contributed to conceptualisation, critical review and editing and approved the final version.

## Funding

Gail Garvey is supported by a National Health and Medical Research Council Investigator Grant (GNT2034453). This project was supported by a Medical Research Future Fund Grant gEnomics4newborns (GNT2015965). The funders had no direct or indirect role in the conception of the study design, data synthesis and interpretation or manuscript writing.

## Disclosure

The lead author used ChatGPT 5.2 (OpenAI) for language editing only. All substantive content, analysis and conclusions were developed by the authors, who take full responsibility for the manuscript. Not commissioned; externally peer reviewed.

## Conflicts of Interest

Sarah Norris is currently the Chair of the Medical Services Advisory Committee (MSAC) Evaluation Sub‐Committee and Deputy Co‐Chair of MSAC.

## Supporting information


**Data S1:** CONSIDER Statement.

## Data Availability

The authors have nothing to report.
