# Supplementary material for: Newborn Screening: Equity for Aboriginal and Torres Strait Islander Families in the Context of Emerging Genomics
Source: Med J Aust. 2026 Sep 2;224(9):e70272. doi: 10.5694/mja2.70272 (PMC13537915; doi:10.5694/mja2.70272)
Supplement: Supplementary file 1 [file MJA2-224-0-s001.pdf]

Supporting Information

Supplementary material

This appendix was part of the submitted manuscript and has been peer reviewed.
It is posted as supplied by the authors.

Appendix to: Garvey G, Norris S, Scarfe J, Lyons L. Newborn screening: equity for Aboriginal and Torres Strait Islander families in the context of emerging genomics. *Med J Aust* 2026; doi: 10.5694/mja2.70272.

CONSIDER Statement

**Governance**
A Project Advisory Group, comprising Aboriginal and Torres Strait Islander representatives, clinicians and researchers, was established to provide cultural governance, strategic oversight, and guidance throughout all phases of the larger project (gEnomics4newborns). The first and senior authors collectively provided cultural oversight and direction in the development of this article.
**Prioritization**
This Perspective article highlights a health priority area for Aboriginal and Torres Strait Islander communities; newborn bloodspot screening (NBS). NBS facilitates early diagnosis and allows treatment and support to be provided for those with screened conditions and improves outcomes for the child and their family. This aligns with Closing the Gap by advocating for culturally safe access and better early childhood health. We draw attention to the key factors to consider in the program to ensure it is culturally safe and accessible for Aboriginal and Torres Strait Islander peoples.
**Relationships (Indigenous stakeholders/participants and Research Team**
Our team acknowledges the importance of reflexively considering and describing our own backgrounds, perspectives and values that we each contribute to the project. The first author (GG) is a proud Kamilaroi woman and senior Aboriginal researcher, and the senior author (LL) is a proud Jaadwa woman and Traditional Owner from Western Victoria. The first and senior authors have expertise and experience in Aboriginal health, research and genomics. SN is a non-Indigenous researcher and brings expertise in health technology assessment and health policy, and JS is a non-Indigenous health economist and health services researcher.
**Methodologies**
This Perspective article was informed by the authors' collective expertise and interpretation of the literature.
**Participation**
This initial article was drafted by the first author and all authors revised and contributed to the manuscript.
**Capacity**
This Perspective article intends to increase awareness of the importance of culturally safe NBS for Aboriginal and Torres Strait Islander families and highlights the need for Aboriginal and Torres Strait Islander leadership, governance, and workforce development in future program design and implementation.
**Analysis and interpretation**
The first and senior authors collectively provided cultural oversight and direction in the development of this perspective article, ensuring that its content and framing were informed by Aboriginal and Torres Strait Islander perspectives and priorities.
**Dissemination**
Published in the Medical Journal of Australia, the article will reach a national audience of clinicians, researchers, policymakers, and health service leaders, drawing attention to the need for equitable implementation of NBS across Australia.

Ethical Research Statement

This perspective article aligns with the CONSIDER statement. Indigenous leadership was central to the development of this article. The first author (GG) is a proud Kamilaroi woman and senior Aboriginal researcher, and the senior author (LL) is a proud Jaadwa woman and Traditional Owner from Western

Victoria. The first and senior authors have expertise and experience in Aboriginal health, research and genomics and collectively they provided cultural oversight and direction in the development of this article. The authors are supported and guided by advice provided by the gEnomics4newborns Aboriginal and Torres Strait Islander Advisory Group.

The initial article was drafted by the first author (GG) and all authors revised and contributed to the manuscript. This article highlights a health priority area for Aboriginal and Torres Strait Islander communities; newborn bloodspot screening (NBS). We draw attention to the key factors to consider in the program to ensure it is culturally safe and accessible for Aboriginal and Torres Strait Islander peoples. This article also draws attention to a future where genomics might be used in NBS and the critical window of opportunity to understand and prioritise the perspectives, hopes, and fears of Aboriginal and Torres Strait Islander peoples regarding the utility of genomics in NBS.
